# Contribution of Mature Hepatocytes to Biliary Regeneration in Rats with Acute and Chronic Biliary Injury

**DOI:** 10.1371/journal.pone.0134327

**Published:** 2015-08-26

**Authors:** Ya-Hui Chen, Hui-Ling Chen, Chin-Sung Chien, Shang-Hsin Wu, Yi-Tian Ho, Chun-Hsien Yu, Mei-Hwei Chang

**Affiliations:** 1 Department of Pediatrics, Taipei Tzu-Chi Hospital, The Buddhist Tzuchi Medical Foundation, Taipei, Taiwan; 2 Department of Pediatrics, Buddhist Tzu-Chi University College of Medicine, Hualien, Taiwan; 3 Graduate Institute of Clinical Medicine, National Taiwan University Hospital and National Taiwan University College of Medicine, Taipei, Taiwan; 4 Hepatitis Research Center, National Taiwan University Hospital and National Taiwan University College of Medicine, Taipei, Taiwan; 5 Department of Pediatrics, National Taiwan University Hospital and National Taiwan University College of Medicine, Taipei, Taiwan; Institute of Hepatology, Foundation for Liver Research, UNITED KINGDOM

## Abstract

Whether hepatocytes can convert into biliary epithelial cells (BECs) during biliary injury is much debated. To test this concept, we traced the fate of genetically labeled [dipeptidyl peptidase IV (DPPIV)-positive] hepatocytes in hepatocyte transplantation model following acute hepato-biliary injury induced by 4,4’-methylene-dianiline (DAPM) and D-galactosamine (DAPM+D-gal) and in DPPIV-chimeric liver model subjected to acute (DAPM+D-gal) or chronic biliary injury caused by DAPM and bile duct ligation (DAPM+BDL). In both models before biliary injury, BECs are uniformly DPPIV-deficient and proliferation of DPPIV-deficient hepatocytes is restricted by retrorsine. We found that mature hepatocytes underwent a stepwise conversion into BECs after biliary injury. In the hepatocyte transplantation model, DPPIV-positive hepatocytes entrapped periportally proliferated, and formed two-layered plates along portal veins. Within the two-layered plates, the hepatocytes gradually lost their hepatocytic identity, proceeded through an intermediate state, acquired a biliary phenotype, and subsequently formed bile ducts along the hilum-to-periphery axis. In DPPIV-chimeric liver model, periportal hepatocytes expressing hepatocyte nuclear factor-1β (HNF-1β) were exclusively DPPIV-positive and were in continuity to DPPIV-positives bile ducts. Inhibition of hepatocyte proliferation by additional doses of retrorsine in DPPIV-chimeric livers prevented the appearance of DPPIV-positive BECs after biliary injury. Moreover, enriched DPPIV-positive BEC/hepatic oval cell transplantation produced DPPIV-positive BECs or bile ducts in unexpectedly low frequency and in mid-lobular regions. These results together suggest that mature hepatocytes but not contaminating BECs/hepatic oval cells are the sources of periportal DPPIV-positive BECs. We conclude that mature hepatocytes contribute to biliary regeneration in the environment of acute and chronic biliary injury through a ductal plate configuration without the need of exogenously genetic or epigenetic manipulation.

## Introduction

The liver has an enormous capacity to regenerate after injury [1–3]. In most situations, liver regeneration is mediated by self duplication of mature hepatocytes and biliary epithelial cells (BECs). Transdifferentiation of hepatocytes and BECs to each other has been proposed as an alternative rescue mechanism in liver diseases when either cell type fails to regenerate by itself [4,5]. It is generally accepted that BECs from canals of Hering proliferate to generate oval cells which can differentiate into hepatocytes when proliferation of hepatocytes is inhibited or overwhelmed [6–9]. However, cell conversion of mature hepatocytes in the opposite direction into BECs during biliary injury is much debated [4,5,10–18].

Several major assays have been used to document the transdifferentiation potential of hepatocytes: in vitro clonogenic growth, cell transplantation, forced activation of Notch signaling, and in vivo lineage tracing [10–19]. In vitro studies have shown that mature hepatocytes can convert into BECs in the organoid culture systems [10,14]. Experiments using hepatocyte transplantation into the spleen have shown that transplanted hepatocytes in the spleen could transdifferentiate into biliary cells that aggregate to form ductular structures [12,15]. Forced activation of Notch signaling can reprogram hepatocytes into BECs in mice [17,19]. Although these studies provide persuasive data, recent in vivo hepatocyte fate tracing studies using genetic lineage tagging or rodents with chimeric livers in chronic biliary liver diseases reach different conclusions [11,13,16–18]. While some studies have shown that hepatocytes undergo widespread hepatocyte-to-BEC reprogramming following bile duct ligation (BDL) or 3,5-diethoxycarbonyl-1,4-dihydrocollidine (DDC) diet induced injury [13,17,18], the others have found no evidence that chronic biliary injury caused by BDL or DDC diet induces conversion of hepatocytes into BECs [11,16].

Cell proliferation is thought to facilitate cell type conversions [20]. Transplanted hepatocytes can proliferate for several rounds to regenerate a damaged liver [21]. Therefore, hepatocyte transplantation experiments would be an ideal model to test the cell-type conversion potential of hepatocytes [16]. However, transplanted hepatocytes have been shown to convert into BECs in the spleen but never in the acute injured liver [12,13,15,22]. In contrast, fetal hepatoblasts and hepatic oval cells have been shown to differentiate as hepatocytes and bile duct cells after transplantation into the damaged liver but not in the spleen [11,12,15,23–25]. We have recently used in vivo lineage tracing technique in rats and resolved the debate on the lineage relationship between mature hepatocytes and small hepatocyte-like progenitor cells (SHPCs) in retrorsine-exposed rats after partial hepatectomy [26]. We have demonstrated that mature hepatocytes do not give rise to SHPCs. Taken together, these and our studies prompted us to hypothesize that being a terminal differentiated cell type, mature hepatocytes can not convert into BECs in the damaged livers. To test this hypothesis, we traced the fate of genetically labeled (DPPIV-positive) hepatocytes in hepatocyte transplantation model following acute hepato-biliary injury induced by 4,4’-methylene-dianiline (DAPM) and D-galactosamine (DAPM+D-gal) and in DPPIV-chimeric liver model subjected to acute hepato-biliary injury (DAPM+D-gal) or chronic biliary injury caused by DAPM and bile duct ligation (DAPM+BDL). In the livers of DPPIV-deficient rats and DPPIV-chimeric livers before biliary injury, BECs are uniformly DPPIV-deficient and proliferation of DPPIV-deficient hepatocytes is restricted by retrorsine. Transplanted hepatocytes and about half of the hepatocytes in the DPPIV chimeric liver are DPPIV-positive and capable of proliferation. If mature hepatocytes were able to transdifferentiate into BECs after biliary injury, we expected to observe DPPIV-positive BECs in the regenerating livers.

## Materials and Methods

### Animals

DPPIV-deficient F344 rats were kindly provided by Professor Sanjeev Gupta from the Albert Einstein College of Medicine. Male DPPIV-deficient rats were used as recipient animals. Normal male DPPIV-positive F344 rats (aged 8–10 weeks, 200–250 g) were purchased from National Laboratory Animal Center, Taiwan and used as donor animals. These animals were in-house bred and maintained on standard laboratory chow and daily 12 hour light/dark cycles. All of the animals received humane care in compliance with the guidelines of the National Science Council of Taiwan (NSC, 1997). All animal experiments were approved by the Institutional Laboratory Animal Care and Use Committee of the National Taiwan University, College of Medicine and College of Public Health and Institutional Animal Care and Use Committee (IACUC), Taipei Tzuchi Hospital, The Buddhist Tzu Chi Medical Foundation (Approval Letter No.: IACUC-20110387, IACUC-20120461, 101-IACUC-001, 101-IACUC-018, and 101-IACUC-033).

### Retrorsine administration, induction of acute hepato-biliary injury, and hepatocyte isolation and transplantation

The retrorsine (Sigma, St. Louis, MO), 4,4’-methylene-dianiline (DAPM) (Sigma, St. Louis, MO), and D-galactosamine (D-gal) (Sigma, St. Louis, MO) working solutions were prepared as described previously and used immediately after preparation [21,22,26,27]. In situ liver perfusion, collagenase digestion, and differential centrifugation were used to purify the hepatocytes from normal male DPPIV-positive F344 rats as previously described [22]. The viability and purity of each preparation were assessed by trypan blue exclusion in a hemacytometer. Preparations typically contained >90% viable hepatocytes.

**Fig 1:**
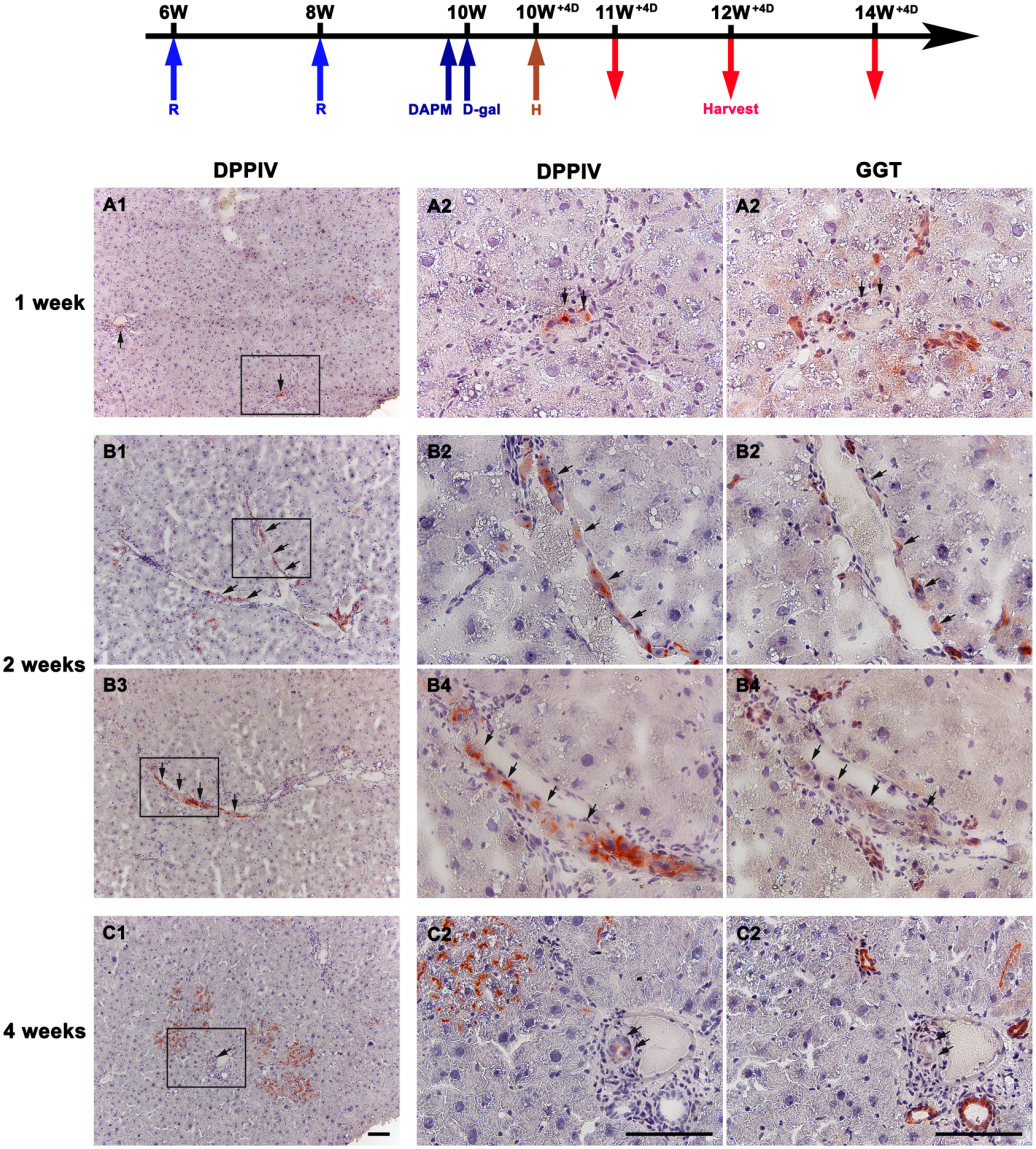
Transplanted DPPIV-positive hepatocytes convert into biliary epithelial cells (BECs) in livers with acute hepato-biliary injury. Shown are scheme illustrating hepatocyte transplantation in retrorsine+DAPM+D-galactosamine treated rats and serial liver sections stained histochemically for DPPIV and Gamma-glutamyl-transpeptidase (GGT, a marker of BECs). (A) At one week, DPPIV-positive hepatocytes (arrows) are in small clusters or short string of 3–5 cells mostly located in the periportal areas. None of the DPPIV-positive cells are GGT-positive. (B) At two weeks, DPPIV-positive cells (arrows) arranged linearly along the portal veins are observed in 30–40% of portal triads per lobe. Some of the linearly arranged DPPIV-positive cells are stained faintly for GGT. (C) At four weeks, DPPIV-positive ducts (arrows) with faint GGT staining in portal triads are found. Middle and right columns are a high magnification view of the area enclosed by the rectangle in left column. (Original magnification: left column,100x; middle and right columns, 400x) Scale bars: 100 μm.

Male DPPIV-deficient rats received two treatments of retrorsine (30 mg/kg, i.p.) two weeks apart, at six and eight weeks of age. D-galactosamine (0.7 g/kg, i.p.) or DAPM (50 mg/kg, i.p.)/D-galactosamine (0.7 g/kg, i.p., 24 hours after DAPM) were used to induce acute hepatic injury (R+D-gal) or acute hepato-biliary injury (R+DAPM+D-gal) two weeks after the second retrorsine treatment. A long-term cannulation of the main portal vein was implanted one week before induction of acute hepato-biliary injury [28]. The rats consciously received intraportal DPPIV-positive hepatocyte transplantation (1x10^7^/ml) through the portal cannula 1 day or 4 days after D-galactosamine treatment. The rats were left to recover and were sacrificed at 1, 2, and 4 weeks after cell transplantation (Fig 1).

### Generation of rats with DPPIV chimeric livers

The rats with DPPIV chimeric livers were generated according to our previous studies [22]. Retrorsine-exposed DPPIV-deficient rats received single injection of D-galactosamine (0.7 g/kg, i.p.) and DPPIV-positive hepatocyte transplantation (1x10^7^/ml) intraportally 24 hours after D-galactosamine treatment. The rats were left to recover for the next one month. In the DPPIV-chimeric livers, hepatocytes derived from the donor DPPIV-positive cells appear positive for DPPIV, whereas host hepatocytes and bile ductules are uniformly negative (S1 Fig). DPPIV-positive hepatocytes comprised about half of the chimeric liver mass at 1 month.

### Isolation and characterization of hepatic oval cells

Hepatic oval cells were isolated from D-galactosamine treated wild-type male F344 rats at day 5 after D-galactosamine treatment [25]. Livers at this time point show the highest number of hepatic oval cells [29,30]. In situ liver perfusion, collagenase digestion, and differential centrifugation are used to purify the hepatic oval cells as described by Yovchev et al. [25]. The viability and purity of each preparation are assessed by trypan blue exclusion in a hemacytometer. Preparations typically contained >95% viable cells. The isolated hepatic oval cells are identified by the labeling of cells with CK-19 antibody and histochemical staining for Gamma-glutamyl-transpeptidase (GGT) to estimate their fractions [25].

### Induction of acute hepato-biliary injury and chronic biliary injury in rats with DPPIV chimeric livers

The rats with DPPIV chimeric livers were randomized to receive one of the following: (1) DAPM (50 mg/kg, i.p.)/D-galactosamine (0.7 g/kg, i.p., 24 hours after DAPM) treatment (Chimeric liver+DAPM+D-gal), (2) DAPM (50 mg/kg, i.p.)/bile duct ligation (BDL, 24 hours after DAPM) treatment (Chimeric liver+DAPM+BDL), (3) two intraperitoneal injection of retrorsine (30 mg/kg) two week apart, followed by DAPM (50 mg/kg, i.p.)/D-galactosamine (0.7 g/kg, i.p., 24 hours after DAPM) treatment (Chimeric liver+R+DAPM+D-gal), or (4) two intraperitoneal injection of retrorsine (30 mg/kg) followed by DAPM (50 mg/kg, i.p.)/BDL treatment (Chimeric liver+R+DAPM+BDL). The rats were allowed to recover and were sacrificed at the indicated time points.

### Histochemistry and Immunohistochemistry

All histochemical and immunohistochemical stainings were performed according to previously described protocols. Primary antibodies are listed in Table 1. Sections of 6 μm thickness were used for histological analysis. DPPIV expression was determined by enzyme histochemical staining in liver cryosections as previously described [31]. Gamma-glutamyl-transpeptidase (GGT) was detected by the method of Rutenberg et al. [32]. Double immunofluorescence stainings were detected using the method described by Paku et al. [33]. Additional methods are provided in the Supporting Information. Appropriate secondary antibodies used in various experiments included Alexa Fluor 488 donkey anti-mouse IgG (Molecular Probes, Oregon, USA) and Alexa Fluor 594 donkey anti-goat IgG (Molecular Probes, Oregon, USA). Nuclei were labeled with 4’,6-diamidino-2-phenylindole (DAPI) (Molecular Probes, Oregon, USA).

**Table 1:** Primary antibodies.

Antibodies	Company/Producer	Cat. number	Dilution
C/EBP-α	Santa Cruz	SC-61	1:200
CFTR	Santa Cruz	SC-10747	1:50
CK-19	Novacastra, Newcastle	NCL-CK19	1:100
CK-7	Santa Cruz	SC-23876	1:150
CPSI	Santa Cruz	SC-10516	1:100
DPPIV	R&D systems	AF954	1:100
HNF-1β	Santa Cruz	SC-22840	1:200
HNF-4α	Santa Cruz	SC-6556	1:50
Laminin	Dako Cytomation	Z0097	1:1000
Lgr5	Novus Biologicals USA	NLS1236	1:200
OV6	R&D systems	MAB2020	1:1000
Sox9	Sigma	HPA001758	1:350
Vimentin	Santa Cruz	SC-6260	1:200

## Results

### Characteristics of acute hepato-biliary injury caused by retrorsine+DAPM+ D-galactosamine (R+DAPM+D-gal) treatment

The combination of retrorsine, DAPM, and D-galactosamine was used to induce acute hepato-biliary injury in DPPIV-deficient rats. In our previous study, retrorsine+D-galactosamine treatment (R+D-gal) causes acute hepatic injury and induced oval cell response [22,30]. The oval cell response emerged at day 1, peaked at days 4–5, and declined after one week [30]. DAPM is a selective biliary toxin [27]. R+DAPM+D-gal treatment caused destruction of biliary trees revealing as ductular swelling and necrosis of biliary epithelium, hepatocellular necrosis, fatty change, inflammatory cell infiltration, and expansion of non-parenchymal epithelial cells at 1 day. At 4 days, the bile ducts appeared to repair from injury, containing new biliary epithelium. The recovery from R+DAPM+D-gal-induced acute hepatobiliary injury was still underway at 4 week. In terms of the oval cell response, the number of oval cells was dramatically suppressed as compared to the peak oval cell response at day 4–5 in R+D-gal treatment, and started to expand at day 7. At week 4, portal triads contained remarkable oval cell ductules extending into the liver lobules (n = 3 rats at each time point) (S2 Fig). Based on these findings, we chose to perform hepatocyte transplantation at day 1 after R+DAPM+D-gal when the rats were in maximal hepatobiliary injury or day 4 when the bile ducts appeared to repair from injury. However, hepatocyte transplantation performed at day 1 after R+DAPM+D-gal caused all mortality of recipient rats in the first two days after transplantation. Intraportal hepatocyte transplantation can cause portal hypertension and result in ischemia/reperfusion injury [28], which might aggravate the hepato-biliary injury in already failing livers at day 1. Only rats receiving hepatocyte transplantation at day 4 after R+DAPM+D-gal were analyzed at 1, 2, and 4 weeks after cell transplantation. Four to five rats were studied for each time point.

### Transplanted DPPIV-positive hepatocytes give rise to biliary epithelial cells in livers with acute hepato-biliary injury

Liver tissues removed at 1, 2, and 4 weeks after hepatocyte transplantation were stained for DPPIV and GGT (a marker of BECs) [29] in serial sections to follow the fate of transplanted hepatocytes. At one week, DPPIV-positive hepatocytes were in small clusters or short string of 3–5 cells mostly located in the periportal areas. None of the DPPIV-positive cells were GGT-positive (Fig 1A). At two weeks, DPPIV-positive hepatocyte clusters were enlarged in size. Notably, DPPIV-positive cells arranged linearly along the portal veins were observed in 30–40% of portal triads per lobe. Some of the linearly arranged DPPIV-positive cells were stained faintly for GGT (Fig 1B). At four weeks, DPPIV-positive cells composed 20.2±8.8% of the liver areas. Unexpectedly, in addition to linearly arranged DPPIV-positive cells along the portal veins, DPPIV-positive ducts with faint GGT staining in portal triads were found (Fig 1C). 1.3±0.6% of DPPIV-positive cells was stained positive for GGT.

**Fig 2:**
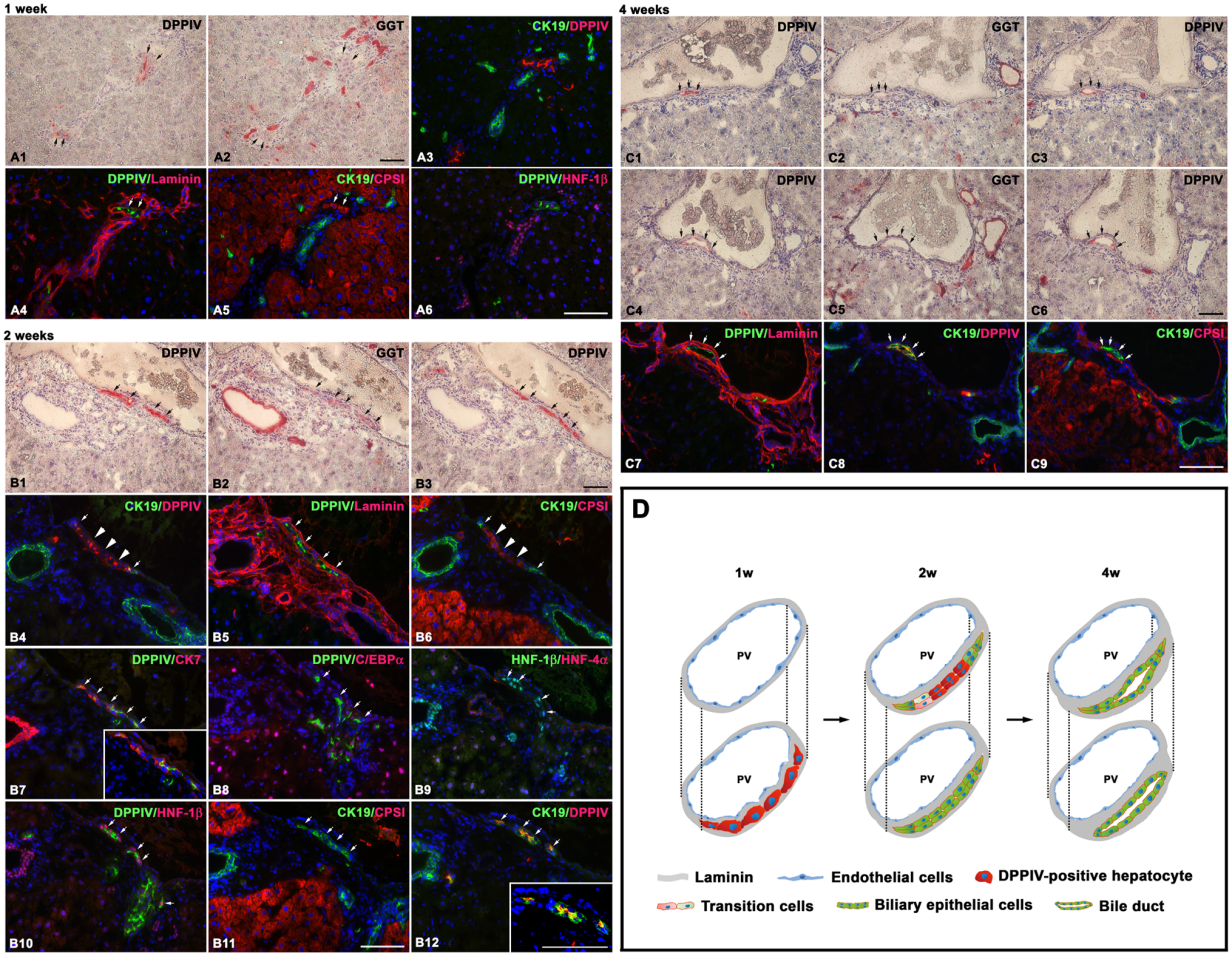
Transplanted DPPIV-positive hepatocytes convert into BECs through a ductal plate configuration in acute hepato-biliary injury. Shown are serial liver sections stained histochemically for DPPIV, GGT, and with double immunofluorescence for DPPIV with laminin, hepatocyte marker (C/EBP-α), BEC markers (CK-19, CK-7, HNF-1β), for CK-19/CPS1 (hepatocyte-specific enzyme), and HNF-1β/HNF-4α (hepatocyte-specific marker). (A1–A6) At one week, periportally entrapped DPPIV-positive cells can be traced in 4–5 sequential sections, are surrounded by laminin, and express only hepatocyte markers (arrows). (B1–B12) At two weeks, the linearly arranged DPPIV-positive cells along the portal veins can be tracked at least in 20 successive sections and are a bilayered structure, indicating that they form a two-layered plate of cells. The plate of DPPIV-positive cells is surrounded by laminin and comprises cells with characteristics of hepatocytes (big arrows), BECs (arrows), and both. A lineage relationship is suggested between these DPPIV-positive cells in the plate, with CPS1(+)/CK-19(-) in some section levels (B4–B6) and CPS1(-), CEBP-α(-)/CK-19(+), CK-7(+), HNF-1β(+) in sequential levels of sections (B7–B12). In some sections, DPPIV-positive plate expressing BEC markers is in contiguity to DPPIV-positive hepatocyte clusters (B10, B11). (C1–C9) At four weeks, most cells of DPPIV-positive plates (arrows) express BEC markers, CK-19(+), CK-7(+), and HNF-1β(+). Lumens are detected focally between the two-layered plates. Serial sections reveal the formation of lumens being along the longitudinal axis of portal veins. (D) Schematic representation of the stepwise conversion of transplanted hepatocytes into BECs. (Original magnification: 200x) Scale bars: 100 μm.

**Fig 3:**
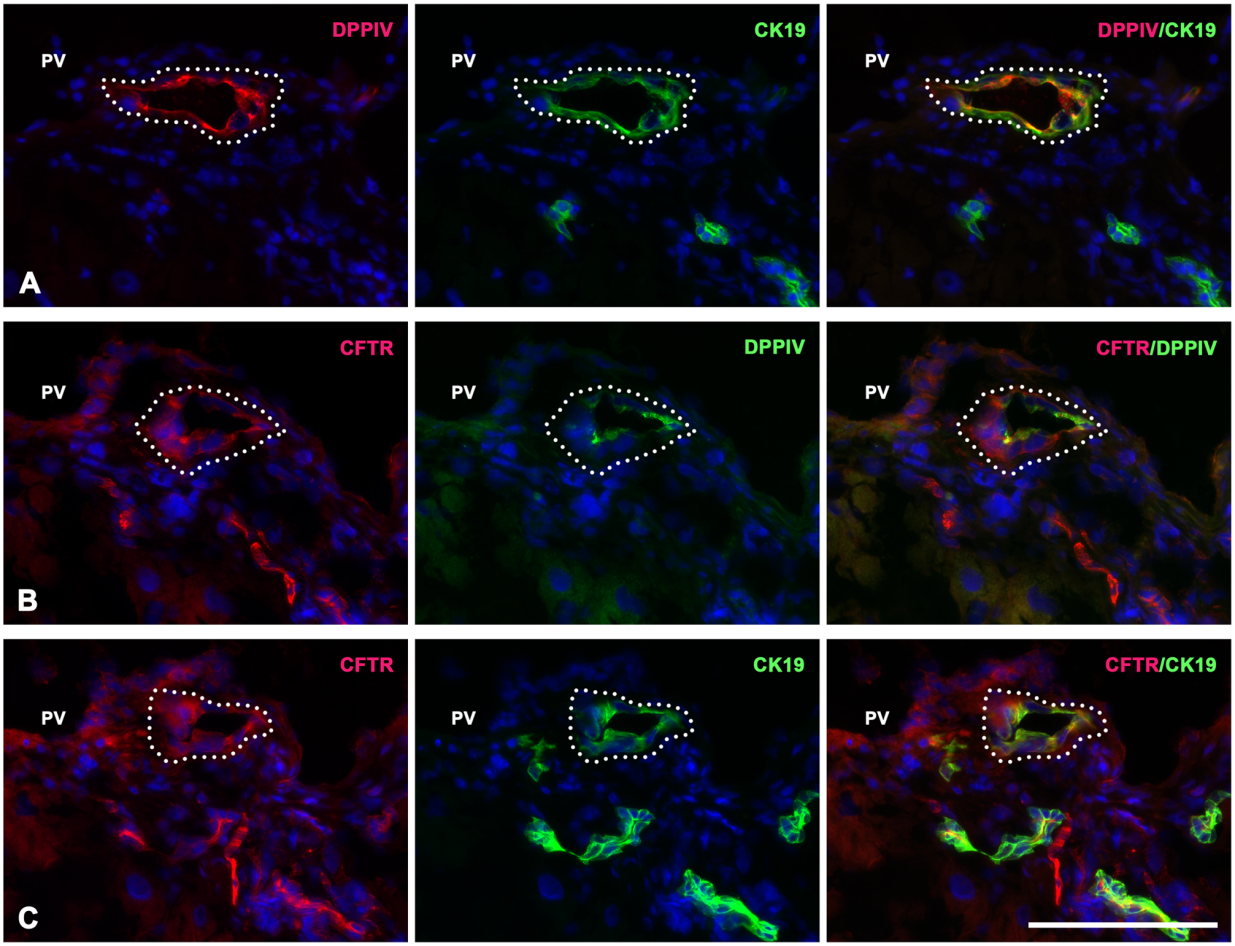
Transplanted DPPIV-positive hepatocytes-derived BECs express a biliary functional apical marker, cystic fibrosis transmembrane regulator (CFTR). Shown are serial liver sections (4 weeks after transplantation) stained with double immunofluorescence for (A) CK-19(green)/DPPIV(red), (B) DPPIV(green)/CFTR(red), and (C) CK-19(green)/CFTR(red). (Original magnification: 400x) Scale bars: 100 μm.

To determine the characteristics of the DPPIV-positive cells arranged in lines or in ducts and their lineage relationship with transplanted DPPIV-positive hepatocytes, we used double immunofluorescence stainings for DPPIV with laminin, hepatocyte marker (CCAAT enhancer binding protein alpha, C/EBP-α) [34], BEC markers (CK-19, CK-7, HNF-1β, Sox9), Lgr5, vimentin, for CK-19/carbamoyl-phosphate-synthetase 1 (CPS1) (hepatocyte-specific enzyme) [35], and HNF-1β/hepatocyte nuclear factor-4α (HNF-4α) (hepatocyte-specific marker) in 40 serial sections at each time point. At one week, the short strings of DPPIV-positive cells in the periportal areas can be followed in 4–5 sequential sections, were surrounded by laminin, and expressed only hepatocyte markers (Fig 2A). At two weeks, the linearly arranged DPPIV-positive cells along the portal veins can be followed at least in 20 successive sections and were a bi-layered structure, indicating that they form a two-layered plate of cells along the longitudinal axis of portal veins. The plate of DPPIV-positive cells was surrounded by laminin and comprised cells with characteristics of hepatocytes, BECs, and both (Fig 2B and S3 Fig). A lineage relationship is suggested between these DPPIV-positive cells in the plate, with CPS1(+)/CK-19(-) in some section levels and CPS1(–) (Fig 2B6 and 2B11), C/EBP-α(-), HNF-4α(-)/CK-19(+), CK-7(+), HNF-1β(+), SRY (sex determining region Y)-box 9 (Sox9)(+) in sequential levels of sections (Fig 2B4, 2B7–2B10 and 2B12, and S3 and S4 Figs). The two-layered plates could be followed back to portal regions. In some sections, DPPIV-positive plate expressing BEC markers was in contiguity to DPPIV-positive hepatocyte clusters (Fig 2B8–2B10). At four weeks, most cells of DPPIV-positive plates expressed BEC markers, CK-19(+), CK-7(+), and HNF-1β(+) (Fig 2C7–2C9 and S3 Fig). Lumens were detected focally between the two-layered plates. Serial sections revealed the formation of lumens being along the longitudinal axis of portal veins (Fig 2C1–2C6). The DPPIV-positive neo-lumens were stained positive for cystic fibrosis transmembrane regulator (CFTR) (Fig 3), a biliary functional marker that is expressed on the apical membrane of bile duct cells [36]. None of the numerous ductular reactions was stained DPPIV(+)/leucine-rich repeat-containing G-protein coupled receptor 5 (Lgr5)(+) or DPPIV(+)/vimentin(+) in all analyzed samples at 1, 2, and 4 weeks (S5 and S6 Figs). These results suggest that transplanted hepatocytes can converse into BECs through a two-layered plate configuration and formation of a neo-lumen in livers with acute hepato-biliary injury (Fig 2D).

### DPPIV-positive hepatocytes convert into BECs in the DPPIV chimeric livers with acute (DAPM+D-gal) and chronic (DAPM+BDL) biliary injuries

**Fig 4:**
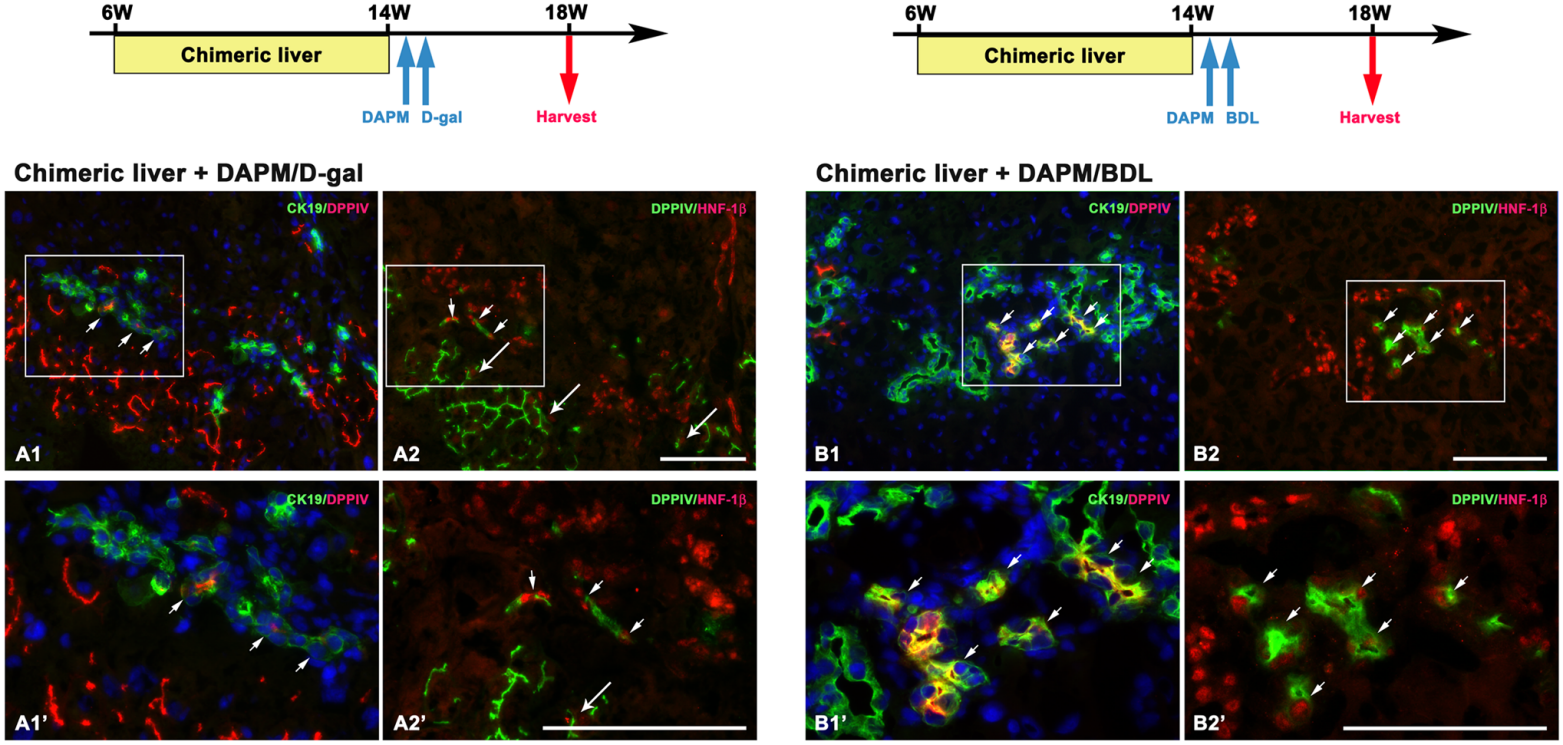
DPPIV-positive hepatocytes convert into BECs in the DPPIV-chimeric livers with acute and chronic biliary injuries. Shown are schemes illustrating chimeric lineage tracing system subjected to acute or chronic biliary injuries and serial liver sections stained with double immunofluorescence for CK-19(green)/DPPIV(red) and DPPIV(green)/HNF-1β(red). At four weeks after biliary injuries, DPPIV-positive BECs [CK-19(+)/DPPIV(+) and DPPIV(+)/HNF-1β(+)] are observed in the portal triads (A1–A2, A1’- A2 ‘, B1–B2, B1’-B2’, arrows). Periportal hepatocytes that express HNF-1β are uniformly DPPIV-positive [CK-19(-)/DPPIV(+) and DPPIV(+)/HNF-1β(+) (long arrows)] and are in continuity to CK-19(+)/DPPIV(+), and DPPIV(+)/HNF-1β(+) BECs, a staining pattern consistent with hepatocyte conversion into BECs. (Original magnification: A1–A2, B1–B2, 200x; A1’-A2’, B1’-B2’, 400x) Scale bars: 100 μm.

Transplanted hepatocytes have been thought to be more amenable to lineage conversion than resident hepatocytes [16]. To corroborate the transdifferentiation potential of mature hepatocytes, we performed the identical acute protocol (DAPM+D-gal) and the chronic biliary injury (DAPM+BDL) in rats with DPPIV-chimeric livers, which harbored endogenous DPPIV-deficient hepatocytes and donor DPPIV-positive hepatocytes [22,26]. At the time of performing acute and chronic biliary injuries, liver histology of DPPIV-chimeric livers was essentially normal. Both DPPIV-deficient and DPPIV-positive hepatocytes were histologically identical, however, the former was proliferation–inhibited and the latter was quiescent [22,26]. As with hepatocyte transplantation model, we observed DPPIV-positive cells expressing biliary-specific markers in the portal areas in both acute hepato-biliary and chronic biliary injury models (n = 4–5 rats at each time point). At four weeks after injuries when biliary restoration was still underway, serial sections co-stained with DPPIV/CK-19 and DPPIV/HNF-1β revealed that DPPIV(+)/CK-19(+) cells expressed strong HNF-1β staining. DPPIV(+)/CK-19(-) hepatocytes immediately adjacent to the DPPIV(+)/CK-19(+) BECs were stained positive for HNF-1β. In contrast, DPPIV(-)/CK-19(-) hepatocytes in the immediate periportal location were never observed to express HNF-1β (Fig 4). The results suggest that the periportal hepatocytes undergo a stepwise conversion into BECs in acute hepato-biliary and chronic biliary injuries. DPPIV-deficient hepatocytes were inhibited to proliferate by retrorsine and thus unable to undergo transdifferentiation [21,22].

### Unambiguous in vivo transdifferentiation of mature hepatocytes

**Fig 5:**
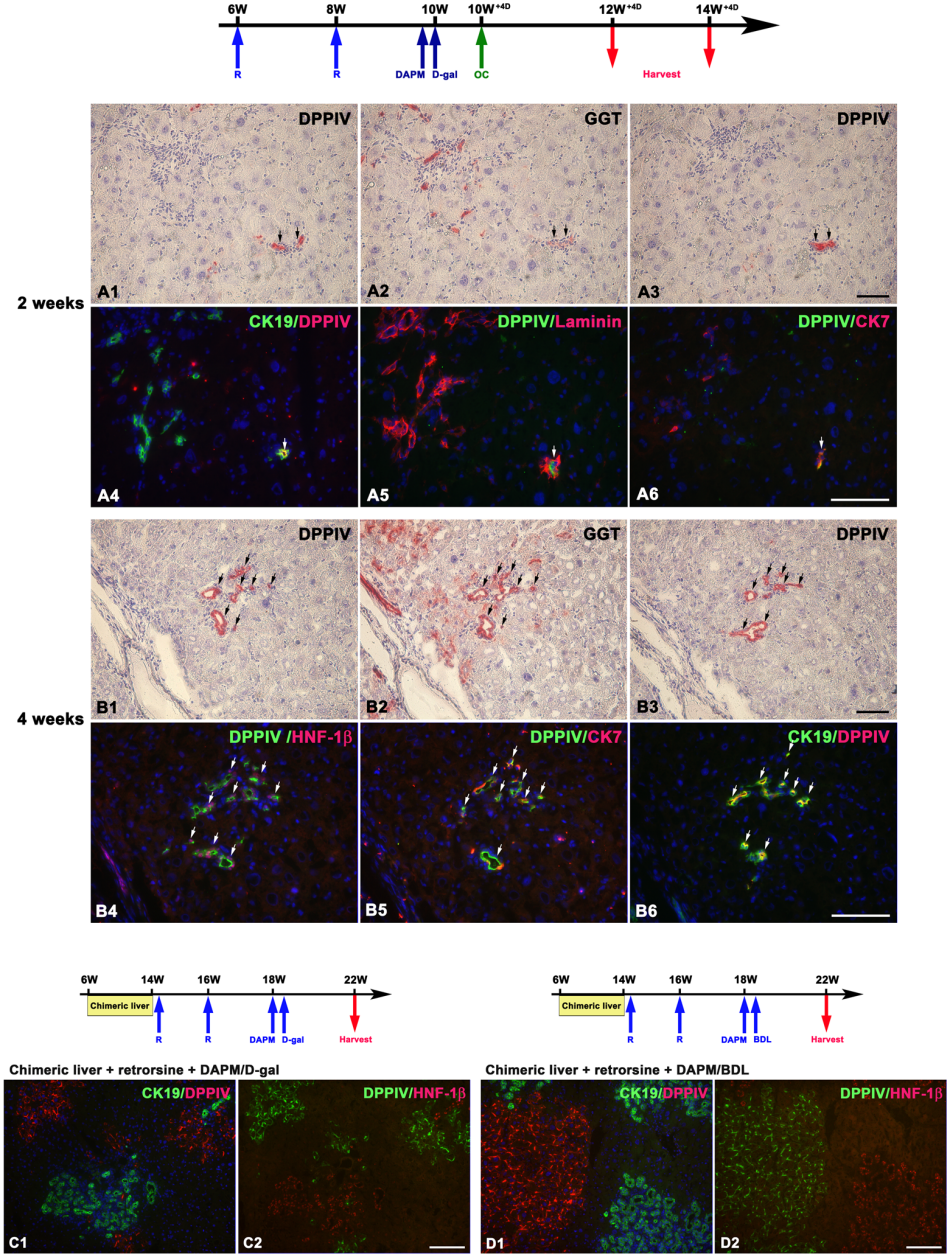
Mature hepatocytes but not contaminating BECs/hepatic oval cells are the sources of periportal DPPIV-positive BECs. (A,B) Shown are scheme illustrating hepatic oval cell transplantation in retrorsine+DAPM+D-galactosamine treated rats and serial liver sections stained histochemically for DPPIV and GGT, and with double immunofluorescence for DPPIV (red) with CK-19 (green), DPPIV (green) with laminin (red) and BEC markers (red) (CK-7, HNF-1β). Transplantation with enriched DPPIV-positive BECs/hepatic oval cells produce DPPIV-positive BECs in the mid-lobular areas in low frequency. (A) At two weeks, DPPIV-positive small cells (arrows) form short two-layered strings in the mid-lobular areas, are GGT(+), CK-19(+), and CK-7(+), and can be tracked in 5–6 sequential sections. (B) At four weeks, DPPIV-positive small cells form multilobulated bile ducts (arrows) in the mid-lobular areas and express strong GGT, HNF-1β, CK-7, CK-19. (C,D) Shown are schemes illustrating chimeric lineage tracing system subjected to retrorsine followed by acute or chronic biliary injuries and serial liver sections stained with double immunofluorescence for CK-19(green)/DPPIV(red) and DPPIV(green)/HNF-1β(red) in DPPIV chimeric livers subjected to retrorsine+DAPM+D-gal and retrorsine+DAPM+BDL at 4 weeks. The numerous regenerating BECs are uniformly DPPIV-deficient. Most DPPIV-positive hepatocyte clusters are located distantly from portal areas. None of the DPPIV-positive hepatocytes express CK-19 and HNF-1β. (Original magnification: A, B, 200x; C, D, 100x) Scale bars: 100 μm.

Although the transplanted hepatocytes we used were highly pure population, it could not be ruled out that contaminating BECs/hepatic oval cells were the sources of DPPIV-positive BECs in the liver after hepatocyte transplantation and injured DPPIV chimeric liver. To test this possibility, we first performed transplantation experiments using enriched BECs/hepatic oval cells populations containing 40–50% of GGT(+)/CK-19(+) cells, that were isolated from wild-type F-344 rats five days after D-galactosamine treatment. We expected to find easily DPPIV-positive BECs. Unexpectedly, however, DPPIV-positive BECs were found only in low frequency, with a variation from none to five per liver lobe, at both two and four weeks after cell transplantation (n = 4–5 rats at each time point) (Fig 5A and 5B). At two weeks, DPPIV-positive small cells formed short two-layered strings in the mid-acinar area, were all GGT(+), CK-19(+), and CK-7(+), and can be followed in 5–6 sequential sections (Fig 5A). At four week, DPPIV-positive small cells formed a single or multilobulated bile ducts and expressed strong GGT, HNF-1β, CK-19 and CK-7 (Fig 5B). This histological appearance was distinct from the hepatocyte-derived BECs that formed ductal plates in the periportal areas and expressed weaker GGT staining.

We next treated rats with DPPIV-chimeric livers with additional two doses of retrorsine to inhibit the proliferation capacity of DPPIV-positive hepatocytes (now DPPIV-positive hepatocytes and DPPIV-deficient hepatocytes were intoxicated with 2 doses and 4 doses of retrorsine, respectively), and then subjected the rats to the two experimental protocols: DAPM+D-gal and DAPM+BDL (n = 4–5 rats at each time point). We expected that the regenerating BECs in these rats with DPPIV-chimeric livers would be uniformly DPPIV-deficient. This was the case. None of the numerous regenerating BECs was stained positive for DPPIV in all analyzed samples from the rats with DPPIV-chimeric livers. Notably, most DPPIV-positive hepatocyte clusters were located distantly from portal areas. Few DPPIV-positive hepatocyte clusters located in the periportal areas, and none of them expressed CK-19 and HNF-1β (Fig 5C and 5D).

These results together support our findings that mature hepatocytes, but not contaminating BECs/hepatic oval cells, are the sources of periportal DPPIV-positive BECs.

## Discussion

In this study, we clearly demonstrate that mature hepatocytes can undergo a stepwise conversion into BECs in the liver during repair from acute hepato-biliary injury and chronic biliary injury. Our findings in DPPIV chimeric livers subjected to chronic biliary injury are consistent with previous studies [13,17,18]. Moreover, our data herein extend the knowledge by showing that mature hepatocytes can give rise to BECs in acute hepato-biliary injury. The hepatocyte transdifferentiation process has some characteristics. First, only mature hepatocytes entrapped in the periportal region participated in the conversion. Second, they proliferated to form two-layered plates and acquired the BEC markers. Third, only a fraction of the two-layered plate cells formed neo-lumens (Fig 2D). Our data confirm the concept that mature hepatocytes have significant phenotypic plasticity [4,5].

In the hepatocyte transplantation experiment in acute hepato-biliary injury model, we observed that the conversion of transplanted DPPIV-positive hepatocytes into BECs is marked by cell proliferation and formation of two-layered plates in the periportal areas. This finding is reminiscent of the ductal plate development observed during embryology [37]. However, biliary differentiation during liver development proceeds through the formation of asymmetrical ductal structures lined on the portal side by cells expressing laminin. Laminin progressively encircles the developing ductal structure, thereby allowing formation of symmetrical bile ducts [37]. We found that transplanted DPPIV-positive hepatocytes entrapped in the periportal regions had been encircled by laminin since the beginning. This may explain why we did not observe asymmetrical ductal structures in the transdifferentiation course. Also, a recent study showed that chronic biliary injury induced ductular metaplasia in both mouse and human mature hepatocytes [38]. Taken together, these findings provide experimental evidence to the concept that ductular reactions in various hepatobiliary diseases have a ductal plate configuration and play a role during postnatally physiological and pathological liver growth [13,39].

However, it is unknown whether the hepatocyte conversion process in chronic biliary injury requires cellular proliferation. Previous studies and our present study have shown that only hepatocytes residing in the periportal areas are able to convert into BECs [13,18,38]. It was reported that periportal hepatocytes of normal livers are hybrid cells expressing dual phenotypes [40]. Recent work showed that hepatocyte-derived ducts revert back to hepatocytes after injury subsides [38]. In addition, it has been shown that the change in composition of extracellular matrix in chronic hepato-biliary injury inhibits hepatocyte proliferation [41]. These studies together suggest that the hepatocyte conversion process in chronic biliary injury may be a switch of phenotypes to avoid insults to hepatocytes with no need of cell proliferation.

We observed that in transplantation experiment or DPPIV-chimeric liver model the conversion of DPPIV-positive hepatocytes into BECs occurred in the periportal regions under acute and chronic biliary injury. This finding is in line with the studies in mice that only those hepatocytes residing around the periportal areas are able to convert into BECs [17,18,38], even though Notch, a signaling pathway mediating biliary programming during liver development, is activated in nearly all hepatocytes [17–19]. These and our studies together suggest that the microenvironment in the periportal areas with biliary injury is critical to the hepatocytes conversion into BECs. It has been shown in the DDC-induced injury model that cells in the periportal areas express an enrichment of genes related to inflammatory response, response to stress, or cell cycle checkpoints [38,42]. The exact molecular mechanisms that govern the hepatocyte transdifferentiation process in the periportal areas remain to be elucidated.

The findings that only a portion of the DPPIV-positive two-layered plate cells participated in the formation of bile ducts and the two-layered plates connected with DPPIV-positive hepatocyte clusters on the parenchymal side reinforce the phenotypic plasticity of hepatocytes. Indeed, hepatocyte dedifferentiation has been shown to be reversible with changes in the composition of the extracellular matrix [42]. Similarly, chronically injured hepatocyte-derived progenitor cells retained a memory of their origin and differentiated back to hepatocytes upon cessation of injury [38].

Despite established transdifferentiation of hepatocytes to BEC in rats with biliary injury in previous studies and ours, the studies in mice using DDC model have generated considerable controversy. In addition, the contribution of hepatocytes to BECs varies largely among studies. The proliferation of host bile ductules is robust in DDC-induced biliary injury in mice and in DAPM-induced biliary injury in rats. Therefore, hepatocytes transdifferentiation may be an injury evasion strategy [38]. We speculate that difference in type, duration, and severity of biliary injury among experimental models may account for the disparity.

It is difficult to estimate the net contribution of mature hepatocytes to biliary regeneration in this study. We can count only DPPIV-positive BECs. However, biliary injury induced by single-dose DAPM was not universal or homogeneous. DPPIV- positive hepatocytes in the transplantation experiment were not exactly entrapped in the injured portal areas. Moreover, DPPIV-positive hepatocytes did not completely occupy the DPPIV chimeric liver mass and uniformly abut on the injured portal region. We may underestimate the numbers of hepatocyte-derived BECs. Our study is more a proof of concept.

DPPIV-positive BECs were not observed in DPPIV chimeric liver treated with additional retrorsine and then subjected to the identical acute and chronic biliary injuries. Both DPPIV-positive and DPPIV-deficient hepatocytes are inhibited to proliferate by retrorsine and are unable to undergo transdifferentiation [26]. Notably, DPPIV-positive hepatocyte clusters in DPPIV chimeric liver were mostly located distantly from portal areas. Our interpretation for this finding is that new DPPIV-deficient hepatocytes might derive from activated host DPPIV-deficient hepatic oval cells and proliferate to push DPPIV-positive hepatocytes toward central veins. This finding would seem to support the old “streaming liver” hypothesis [16].

A basic concern must be addressed is whether contaminating BECs/hepatic oval cells that composed less than 1% in the transplanted hepatocyte population could be the source of DPPIV-positive BECs in the acute injured livers receiving hepatocyte transplantation. Several evidences argue against this possibility. First, transplantation with enriched DPPIV-positive BECs/hepatic oval cells (40–50% of twenty million cells transplanted, 100 times higher than the contaminating number in transplanted hepatocyte population) produced DPPIV-positive BECs or bile ducts in unexpectedly low frequency (0–5 strings or ducts per liver lobe). Second, these DPPIV-positive BECs or bile ducts were located in the mid-lobular zones. Third, they expressed strong GGT and exclusively BEC-specific markers throughout the study time. These characteristics were remarkably distinct from those of DPPIV-positive hepatocyte- derived BECs. The low efficiency with enriched DPPIV-positive BEC/hepatic oval cell transplantation is consistent with previous studies [23–25]. A possible reason for this is that BECs/hepatic oval cells are small in size [11,23–25]. They move easily to reach beyond mid-acinus of the liver lobules and are less efficiently trapped in the liver.

We did not observe DPPIV-positive hepatocytes acquiring mesenchymal morphology during their conversion into BECs based on histopathological analysis. This could be a limitation of this study, since recent work showed that conversion of hepatocytes into BECs was marked by induction of mesenchymal markers in vitro [38]. However, tissue histopathology can provide a wealth of irreplaceable data about structural integrity, spatial and temporal relationships, and rare events/cells [9,40]. In addition, cellular morphology and physiology are prone to change in vitro. Cell conversion would ideally be tested in vivo [20].

GGT expression was weak in DPPIV-positive plates and BECs derived from transplanted DPPIV-positive hepatocytes throughout the study time. This GGT expression pattern further supports their hepatocyte origin instead of hepatic oval cells/BECs origin. GGT expression is driven by several promoters during liver development. Hepatoblasts and hepatic precursor cells lose the promoters that drive GGT expression when they differentiate into hepatocytes [43]. Recent elegant work showed that hepatocyte-derived and biliary-derived bile ducts expressed distinct level of bile duct markers [38].

A recent study by Isse et al. showed that hybrid transitional hepatocytes existed in the periportal area of normal human liver [40]. We cannot exclude the possibility that some hybrid transitional hepatocytes in the transplanted hepatocyte population might form the cell plates and give rise to BECs in this study. However, the number of hybrid hepatocytes in normal liver has not been quantified in human or rodents and should be rare. Transplanted hepatocytes can acquire the position-specific enzyme expression depending on their lobular location [40,44,45]. The transplanted hepatocytes entrapped in the periportal area should have statistically more chance to be mature hepatocytes than hybrid hepatocytes and acquire the BEC-specific markers.

Based on this study, we conclude that mature hepatocytes contribute to the biliary regeneration in the environment of acute and chronic biliary injury through a ductal plate configuration without the need of exogenously genetic or epigenetic manipulation. Our finding should be valuable in developing hepatocyte transplantation therapy for hepato-biliary diseases.

## Supplementary Materials and Methods

### Histochemistry and Immunohistochemistry

All histochemical and immunohistochemical stainings were performed according to previously described protocols. Primary antibodies are listed in Table 1. Sections of 6 μm thickness were used for histological analysis. DPPIV expression was determined by enzyme histochemical staining in liver cryosections as previously described [31]. Gamma-glutamyl-transpeptidase (GGT) was detected by the method of Rutenberg et al. [32]. Double immunofluorescence staining for DPPIV (R&D, Minneapolis, USA) and CK-19 (Novocastra, Newcastle, UK), DPPIV and laminin (DAKO, CA, USA), CK-19 and Carbamoyl-phosphate-synthetase 1 (CPS1) (Santa Cruz Biotechnology, CA, USA), DPPIV and CK-7 (Santa Cruz Biotechnology, CA, USA), DPPIV and C/EBP-α (Santa Cruz Biotechnology, CA, USA), DPPIV and hepatocyte nuclear factor-1β (HNF-1β) (Santa Cruz Biotechnology, CA, USA), DPPIV and Sox9 (Sigma, St. Louis, MO), DPPIV and Lgr5 (Novus Biologicals, USA), DPPIV and vimentin (Santa Cruz Biotechnology, CA, USA), HNF-1β and hepatocyte nuclear factor-4α (HNF-4) (Santa Cruz Biotechnology, CA, USA), CK-19 and Carbamoyl-phosphate-synthetase 1 (CPS1) (Santa Cruz Biotechnology, CA, USA), DPPIV and cystic fibrosis transmembrane regulator (CFTR) (Abcam, Cambridge, MA, USA), and CK-19 and CFTR were detected using the method described by Paku et al. [33]. Appropriate secondary antibodies used in various experiments included Alexa Fluor 488 donkey anti-mouse IgG (Molecular Probes, Oregon, USA) and Alexa Fluor 594 donkey anti-goat IgG (Molecular Probes, Oregon, USA). Nuclei were labeled with 4’,6-diamidino-2-phenylindole (DAPI) (Molecular Probes, Oregon, USA).

## Supporting Information

S1 Fig DPPIV histochemistry of normal wild-type liver, DPPIV-deficient liver, and DPPIV-chimeric liver. (A) Hepatocytes and bile duct epithelial cells are stained red (positive) for DPPIV in the normal Fisher rat liver, the former in a bile canalicular pattern and the latter in a diffuse cytoplasmic expression pattern. (B) Hepatocytes and bile duct epithelial cells are negative for DPPIV staining in the DPPIV-deficient rat liver. (C) Bile canaliculi of donor hepatocytes are stained red for DPPIV, and bile ductules are uniformly negative for DPPIV staining in the DPPIV chimeric liver of DPPIV-deficient rats. (Original magnification: A1, B1, C1, 100x; A2, 400x; B2, C2, 200x; Scale bars: 100 μm.).
(TIF)

S2 Fig Characteristics of acute hepato-biliary injury caused by retrorsine+D-galactosamine (R+D-gal) treatment and retrorsine+DAPM+ D-galactosamine (R+DAPM+D-gal) treatment. Liver sections are analyzed using double immunofluorescence staining for CK-19 (green)/C/EBP-α (red) and OV6 (green)/Sox9 (red) in retrorsine+D-galactosamine treated rats (R+ D-gal, A), and retrorsine+DAPM+D-galactosamine treated rats (R+DAPM+D-gal, B). (Original magnification: 200x; Scale bars: 100 μm.).
(TIF)

S3 Fig Transplanted DPPIV-positive hepatocytes convert into BECs through a ductal plate configuration in acute hepato-biliary injury. Shown are original single color and merged images of Fig 2B7 DPPIV(green)/CK-7(red), 2B9 HNF-1β(green)/HNF-4α(red), 2B10 DPPIV(green)/HNF-1β(red), 2B12 CK-19(green)/DPPIV(red), and 2C8 CK-19(green)/DPPIV(red). (Original magnification: 200x; Scale bars: 100 μm.).
(TIF)

S4 Fig Transplanted DPPIV-positive cells express Sox9 at 2 weeks after hepatocyte transplantation in R+DAPM+D-gal treated liver. Shown are representative figures of double immunofluorescence staining for DPPIV (green)/Sox9 (red) in serial sections in R+DAPM+D-gal treated liver at 2 weeks after hepatocyte transplantation. Transplanted DPPIV-positive cells express Sox9 (arrow) at 2 weeks after hepatocyte transplantation in R+DAPM+D-gal treated liver. (Original magnification: A, 200x; B, C, 400x; Scale bars: 100 μm.).
(TIF)

S5 Fig Transplanted DPPIV-positive cells do not express Lgr5 during their conversion into BECs in R+DAPM+D-gal-treated livers. Shown are representative figures of (A) Lgr5(+) (arrow) staining in normal colon (positive control), (B) dual immunofluorescence staining for CK-19(+)/Lgr5(+) in bile duct ligation liver (BDL) at 3 weeks (positive control), and (C) dual immunofluorescence stainings for CK-19(+)/DPPIV(+) and DPPIV(+)/Lgr5(-) in R+DAPM+D-gal treated liver sections at 2 weeks after hepatocyte transplantation. Transplanted DPPIV-positive cells did not express Lgr5 during their conversion into BECs in R+DAPM+D-gal-treated livers. (Original magnification: A, B, 200x; C, 400x; Scale bars: 100 μm.).
(TIF)

S6 Fig Transplanted DPPIV-positive cells do not express vimentin during their conversion into BECs in R+DAPM+D-gal-treated livers. Shown are representative figures of vimentin staining in bile duct ligation liver (BDL) at 4 weeks (positive control) and of dual immunofluorescence stainings for DPPIV (green)/vimentin (red) in R+DAPM+D-gal treated liver sections at 1 and 2 weeks after hepatocyte transplantation. (Original magnification: A,100x; A’, 200x; B, C, 400x; Scale bars: 100 μm.).
(TIF)

## References

[1] Michalopoulos GK, DeFrances MC. Liver regeneration. Science 1997;276: 60–66. 9082986 10.1126/science.276.5309.60

[2] Fausto N, Campell JS, Riehle KJ. Liver regeneration. Hepatology 2006;43 (Suppl. 1): S45–S53. 16447274 10.1002/hep.20969

[3] Sell S. Heterogeneity and plasticity of hepatocyte lineage cells. Hepatology 2001;33: 738–750. 11230756 10.1053/jhep.2001.21900

[4] Michalopoulos GK. Phenotypic fidelity (or not?) of epithelial cells in the liver. Hepatology 2012;55: 2024–2026. 10.1002/hep.25703 22407729 PMC3365605

[5] Michalopoulos GK. The liver is a peculiar organ when it comes to stem cells. Am J Pathol 2014;184: 1263–1267. 10.1016/j.ajpath.2014.02.020 24681248 PMC4005979

[6] Fausto N, Campbell JS. The role of hepatocytes and oval cells in liver regeneration and repopulation. Mech Dev 2003;120: 117–130. 12490302 10.1016/s0925-4773(02)00338-6

[7] Fausto N. Liver regeneration and repair: hepatocytes, progenitor cells, and stem cells. Hepatology 2004;39: 1477–1487. 15185286 10.1002/hep.20214

[8] Factor VM, Radaeva SA, Thorgeirsson SS. Origin and fate of oval cells in dipin-induced hepatocarcinogenesis in the mouse. Am J Pathol 1994;145: 409–422. 8053498 PMC1887389

[9] Kuwahara R, Kofman AV, Landis CS, Swenson ES, Barendswaard E, Theise ND. The hepatic stem cell niche: identification by label-retaining cell assay. Hepatology 2008;47: 1994–2002. 10.1002/hep.22218 18454509 PMC2847183

[10] Michalopoulos GK, Bowen WC, Mule K, et al Hepatocytes undergo phenotypic transformation to biliary epithelium in organoid cultures. Hepatology 2002;36: 278–283. 12143035 10.1053/jhep.2002.34858 PMC1769334

[11] Wang X.; Foster M.; Al-Dhalimy M.; Lagasse E.; Finegold M.; Grompe M. The origin and liver repopulation capacity of murine oval cells. Proc. Natl. Acad. Sci. U.S.A. 2003;100: 11881–11888. 12902545 10.1073/pnas.1734199100 PMC304102

[12] Fukuda K, Sugihara A, Nakasho K, Tsujimura T, Yamada N, Okaya A, et al The origin of biliary ductular cells that appear in the spleen after transplantation of hepatocytes. Cell Transplant 2004;13: 27–33. 15040602 10.3727/000000004772664860

[13] Michalopoulos GK, Barus L, Bowen WC. Transdifferentiation of rat hepatocytes into biliary cells after bile duct ligation and toxic biliary injury. Hepatology 2005;41: 535–544. 15726663 10.1002/hep.20600 PMC1821079

[14] Nishikawa Y, Doi Y, Watanabe H, et al Transdifferentiation of mature rat hepatocytes into bile duct-like cells in vitro. Am J Pathol 2005;166: 1077–1088. 15793288 10.1016/S0002-9440(10)62328-0 PMC1602375

[15] Watanabe H, Hata M, Terada N, Ueda H, Yamada N, Yamanegi K, et al Transdifferentiation into biliary ductular cells of hepatocytes transplanted into the spleen. Pathology 2008;40: 272–276. 10.1080/00313020801911546 18428047

[16] Malato Y, Naqvi S, Schurmann N, Ng R, Wang B, Zape J, et al Fate tracing of mature hepatocytes in mouse liver homeostasis and regeneration. J Clin Invest 2011;121: 4850–4860. 10.1172/JCI59261 22105172 PMC3226005

[17] Yanger K, Zong Y, Maggs LR, Shapira SN, Maddipati R, Aiello NM, et al Robust cellular reprogramming occurs spontaneously during liver regeneration. Genes Dev. 2013;27(7): 719–724. 10.1101/gad.207803.112 23520387 PMC3639413

[18] Sekiya S, Suzuki A. Hepatocytes, rather than cholangiocytes, can be the major source of primitive ductules in the chronically injured mouse liver. Am J Pathol 2014;184: 1468–1478. 10.1016/j.ajpath.2014.01.005 24594350

[19] Zong Y, Panikkar A, Xu J, Antoniou A, Raynaud P, Lemaigre F, et al Notch signaling controls liver development by regulating biliary differentiation. Development 2009;136: 1727–1739. 10.1242/dev.029140 19369401 PMC2673761

[20] Zhou Q, Melton DA. Extreme makeover: converting one cell into another. Cell Stem Cell 2008;3: 382–388. 10.1016/j.stem.2008.09.015 18940730

[21] Laconi E, Oren R, Mukhopadhyay DK, Hurston E, Laconi S, Pani P, et al Long term, near total liver replacement by transplantation of hepatocytes in rats treated with retrorsine. Am J Pathol 1998;158: 319–329. 10.1016/S0002-9440(10)65574-5 PMC1852941 9665494

[22] Yu CH, Chen HL, Chen YH, Chien CS, Chang MF, Chang MH. Impaired Hepatocyte Regeneration in Acute Severe Hepatic Injury Enhances Effective Repopulation by Transplanted Hepatocytes. Cell Transplant 2009;18: 1081–1092. 10.3727/096368909X12483162196647 19650970

[23] Sandhu JS, Petkov PM, Dabeva MD, Shafritz DA. Stem cell properties and repopulation of the rat liver by fetal liver epithelial progenitor cells. Am J Pathol 2001;159: 1323–1334. 11583960 10.1016/S0002-9440(10)62519-9 PMC1850488

[24] Simper-Ronan R, Brilliant KB, Flanagan D, Carreiro M, Callanan H, Sabo E, et al Cholangiocyte marker-positive and–negative fetal liver cells differ significantly in the ability to regenerate the livers of adult rats exposed to retrorsine. Development 2006;133: 4269–4279. 17021035 10.1242/dev.02589

[25] Yovchev MI, Grozdanov PN, Zhao H, Racherla H, Guha C, Dabeva MD. Identification of adult hepatic progenitor cells capable of repopulating injured rat liver. Hepatology 2008;47: 636–647. 18023068 10.1002/hep.22047

[26] Chen YH, Chang MH, Chien CS, Wu SH, Yu CH, Chen HL. Contribution of mature hepatocytes to small hepatocyte-like progenitor cells in retrorsine-exposed rats with chimeric livers. Hepatology 2013;57: 1215–1224. 10.1002/hep.26104 23080021

[27] Petersen BE, Zajac VF, Michalopoulos GK. Bile duct damage induced by methylene dianiline inhibits oval cell activation. Am J Pathol 1997;151: 905–909. 9327722 PMC1858051

[28] Yu CH, Chen HL, Chen WT, Ni YH, Lin YL, Chang MH. Portal hemodynamic changes after hepatocyte transplantation in acute hepatic failure. J Biomed Sci 2004;11:756–763. 15591772 10.1007/BF02254360

[29] Dabeva M. D.; Shafritz D. A. Activation, proliferation, and differentiation of progenitor cells into hepatocytes in the D-galactosamine model of liver regeneration. Am J Pathol 1993;143: 1606–1620. 7504886 PMC1887261

[30] Yu CH, Chang MH, Chen YH, Chang MF, Chien CS, Chen HL. Hepatocyte transplantation and the differentiation fate of host oval cells in acute severe hepatic injury. Cell Transplant 2010;19: 231–243. 10.3727/096368909X479848 19906331

[31] Rajvanshi P, Kerr A, Bhargava KK, Burk RD, Gupta S. Studies of liver repopulation using the dipeptidyl peptidase IV deficient rats. Hepatology 1996;23: 482–496. 8617428 10.1002/hep.510230313

[32] Rutenburg AM, Kim H, Fischbein JW, Hanker JS, Wassekrug HL, Seligman AM. Histochemical and ultrastructural demonstration of gamma-glutamyl transpeptidase activity. Histochem Cytochem 1969;17: 517–526. 10.1177/17.8.517 5816239

[33] Paku S, Nagy P, Kopper L, Thorgeirsson SS. 2-acetylaminofluorene dose-dependent differentiation of rat oval cells into hepatocytes: confocal and electron microscopic studies. Hepatology 2004;39: 1353–1361. 15122764 10.1002/hep.20178

[34] Lorenzini S, Bird TG, Boulter L, Bellamy C, Samuel K, Aucott R, et al Characterization of a stereotypical cellular and extracellular adult liver progenitor cell niche in rodents and diseased human liver. Gut 2010;59: 645–654. 10.1136/gut.2009.182345 20427399 PMC3034133

[35] Strick-Marchand H, Morosan S, Charneau P, Kremsdorf D, Weiss MC. Bipotential mouse embryonic liver stem cell lines contribute to liver regeneration and differentiate as bile ducts and hepatocytes. Proc Natl Acad Sci USA 2004;101: 8360–8365. 15155906 10.1073/pnas.0401092101 PMC420399

[36] Feranchak AP, Sokol RJ. Cholangiocyte biology and cystic fibrosis liver disease. Semin Liver Dis 2001;21: 471–488. 11745036 10.1055/s-2001-19030

[37] Antoniou A, Raynaud P, Cordi S, Zong Y, Tronche F, Stanger BZ, et al Intrahepatic bile ducts develop according to a new mode of tubulogenesis regulated by the transcription factor SOX9. Gastroenterology 2009;136: 2325–2333. 10.1053/j.gastro.2009.02.051 19403103 PMC2743481

[38] Tarlow BD, Pelz C, Naugler WE, Wakefield L, Wilson EM, Finegold MJ, et al Bipotential adult liver progenitors are derived from chronically injured mature hepatocytes. Cell Stem Cell 2014;15: 605–618. 10.1016/j.stem.2014.09.008 25312494 PMC4254170

[39] Desmet VJ. Ductal plates in hepatic ductular reactions. Hypothesis and implications. II. Ontogenic liver growth in childhood. Virchows Arch 2011;458: 261–270. 10.1007/s00428-011-1049-2 21298286

[40] Isse K, Lesniak A, Grama K, Maier J, Specht S, Castillo-Rama M, et al Preexisting epithelial diversity in normal human livers: a tissue-tethered cytometric analysis in portal/periportal epithelial cells. Hepatology 2013;57(4): 1632–1643. 10.1002/hep.26131 23150208 PMC3612393

[41] Liu L, Yannam GR, Nishikawa T, Yamamoto T, Basma H, Ito R, et al The microenvironment in hepatocyte regeneration and function in rats with advanced cirrhosis. Hepatology 2012;55: 1529–1539. 10.1002/hep.24815 22109844 PMC3700584

[42] Godoy P, Hengstler JG, Ilkavets I, Meyer C, Bachmann A, Muller A, et al Extracellular matrix modulates sensitivity of hepatocytes to fibroblastoid dedifferentiation and transforming growth factor beta-induced apoptosis. Hepatology 2009;49: 2031–2043. 10.1002/hep.22880 19274752

[43] Holic N, Suzuki T, Corlu A, Couchie D, Chobert MN, Guguen-Guillouzo C, et al Differential expression of the rat γ–glutamyl transpeptidase gene promoters along with differentiation of hepatoblasts into biliary or hepatocytic lineage. Am J pathol 2000;157: 537–548. 10934156 10.1016/s0002-9440(10)64564-6 PMC1850145

[44] Koenig S, Aurich H, Schneider C, Krause P, Haftendorn R, Becker H, et al Zonal expression of hepatocytic marker enzymes during liver repopulation. Histochem Cell Biol 2007;128: 105–114. 17576590 10.1007/s00418-007-0301-y

[45] Wu YM, Joseph B, Berishvili E, Kumaran V, Gupta S. Hepatocyte transplantation and drug-induced perturbations in liver cell compartments. Hepatology 2008;47: 279–287. 17935178 10.1002/hep.21937

