# Supplementary figures and images for: Contribution of Mature Hepatocytes to Biliary Regeneration in Rats with Acute and Chronic Biliary Injury
Source: PLoS One. 2015 Aug 26;10(8):e0134327. doi: 10.1371/journal.pone.0134327 (PMC4550468; doi:10.1371/journal.pone.0134327)

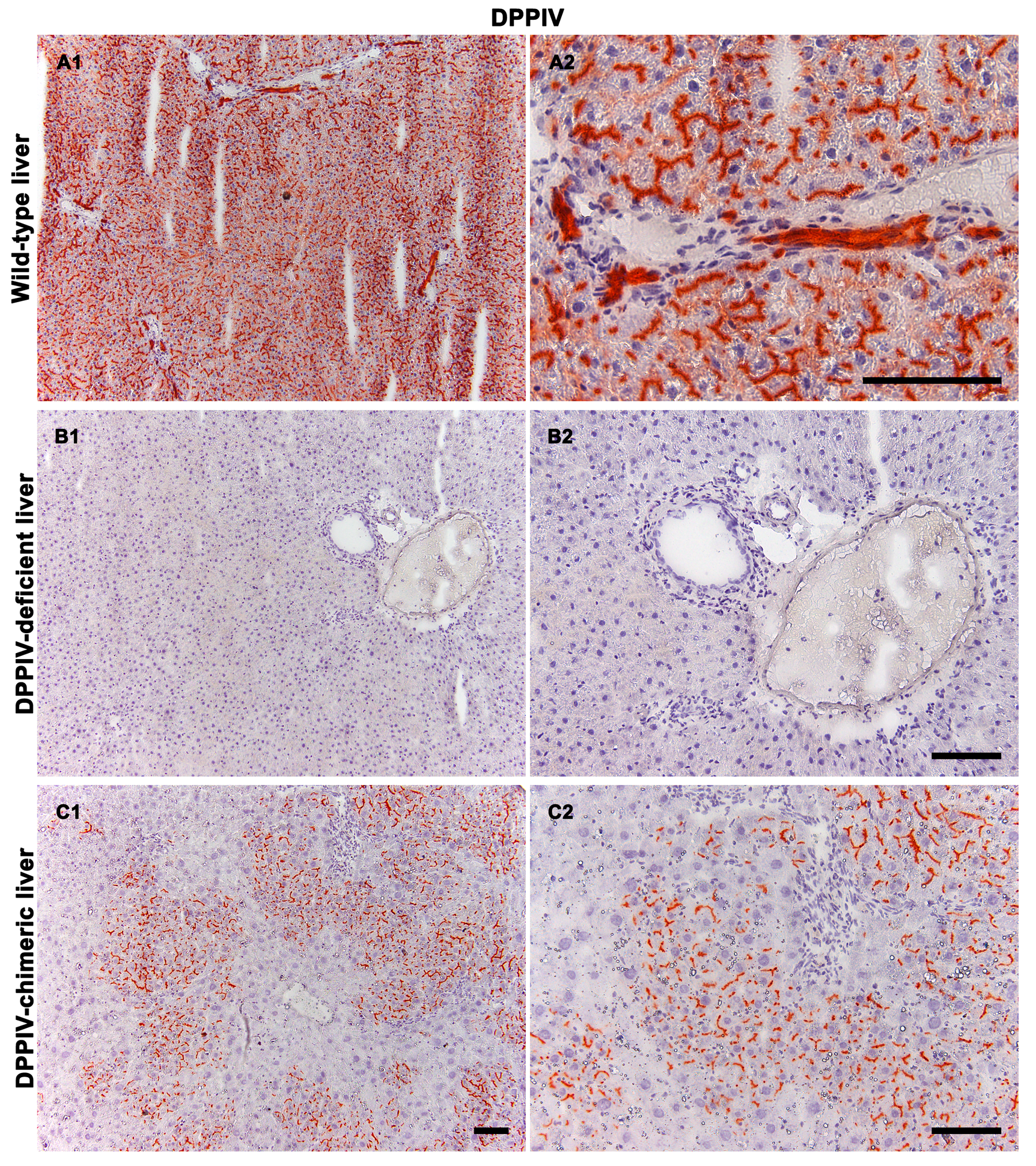

Supplement: S1 Fig — (A) Hepatocytes and bile duct epithelial cells are stained red (positive) for DPPIV in the normal Fisher rat liver, the former in a bile canalicular pattern and the latter in a diffuse cytoplasmic expression pattern. (B) Hepatocytes and bile duct epithelial cells are negative for DPPIV staining in the DPPIV-deficient rat liver. (C) Bile canaliculi of donor hepatocytes are stained red for DPPIV, and bile ductules are uniformly negative for DPPIV staining in the DPPIV chimeric liver of DPPIV-deficient rats. (Original magnification: A1, B1, C1, 100x; A2, 400x; B2, C2, 200x; Scale bars: 100 μm.). (TIF) [file pone.0134327.s001.tif]

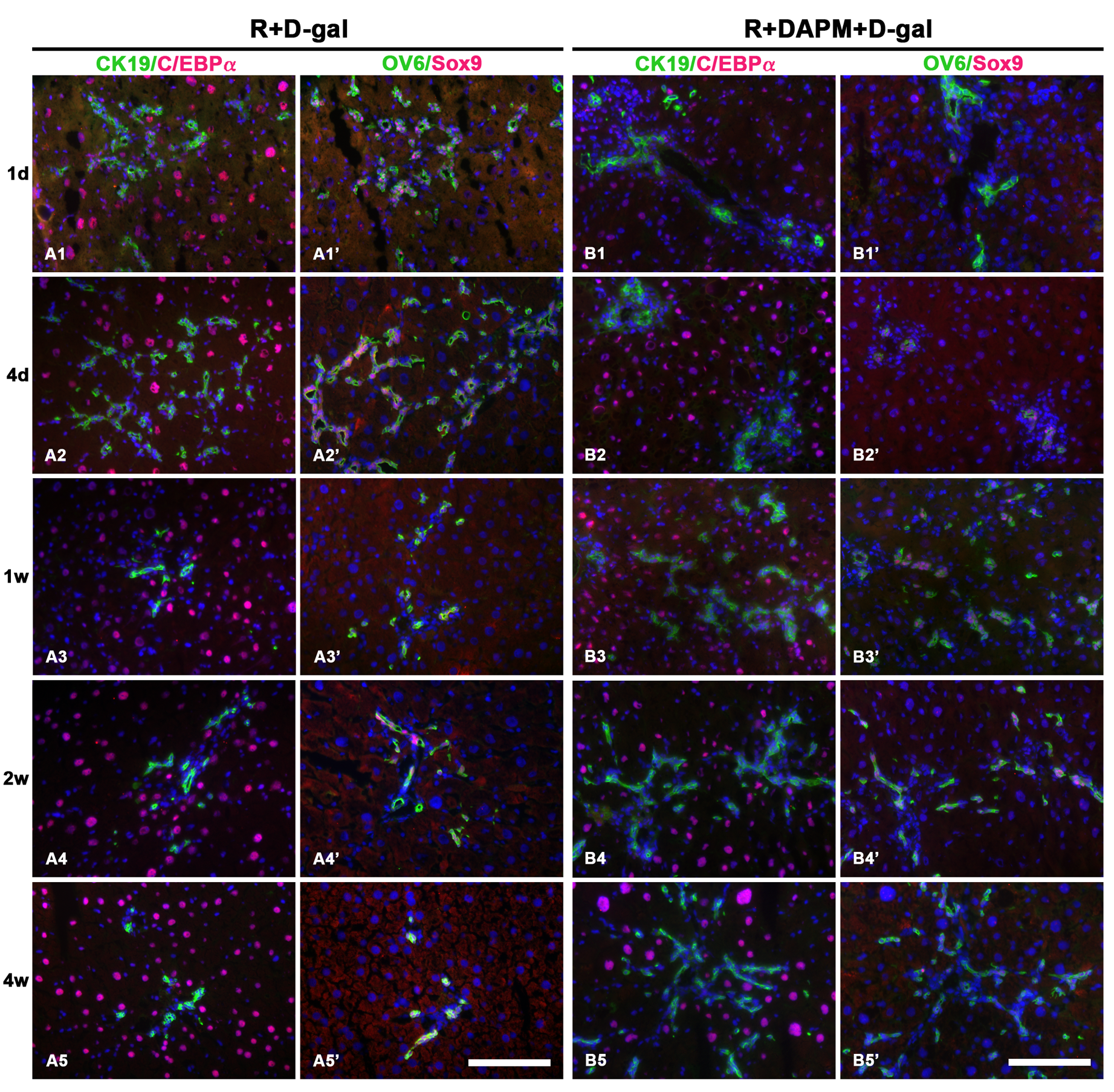

Supplement: S2 Fig — Liver sections are analyzed using double immunofluorescence staining for CK-19 (green)/C/EBP-α (red) and OV6 (green)/Sox9 (red) in retrorsine+D-galactosamine treated rats (R+ D-gal, A), and retrorsine+DAPM+D-galactosamine treated rats (R+DAPM+D-gal, B). (Original magnification: 200x; Scale bars: 100 μm.). (TIF) [file pone.0134327.s002.tif]

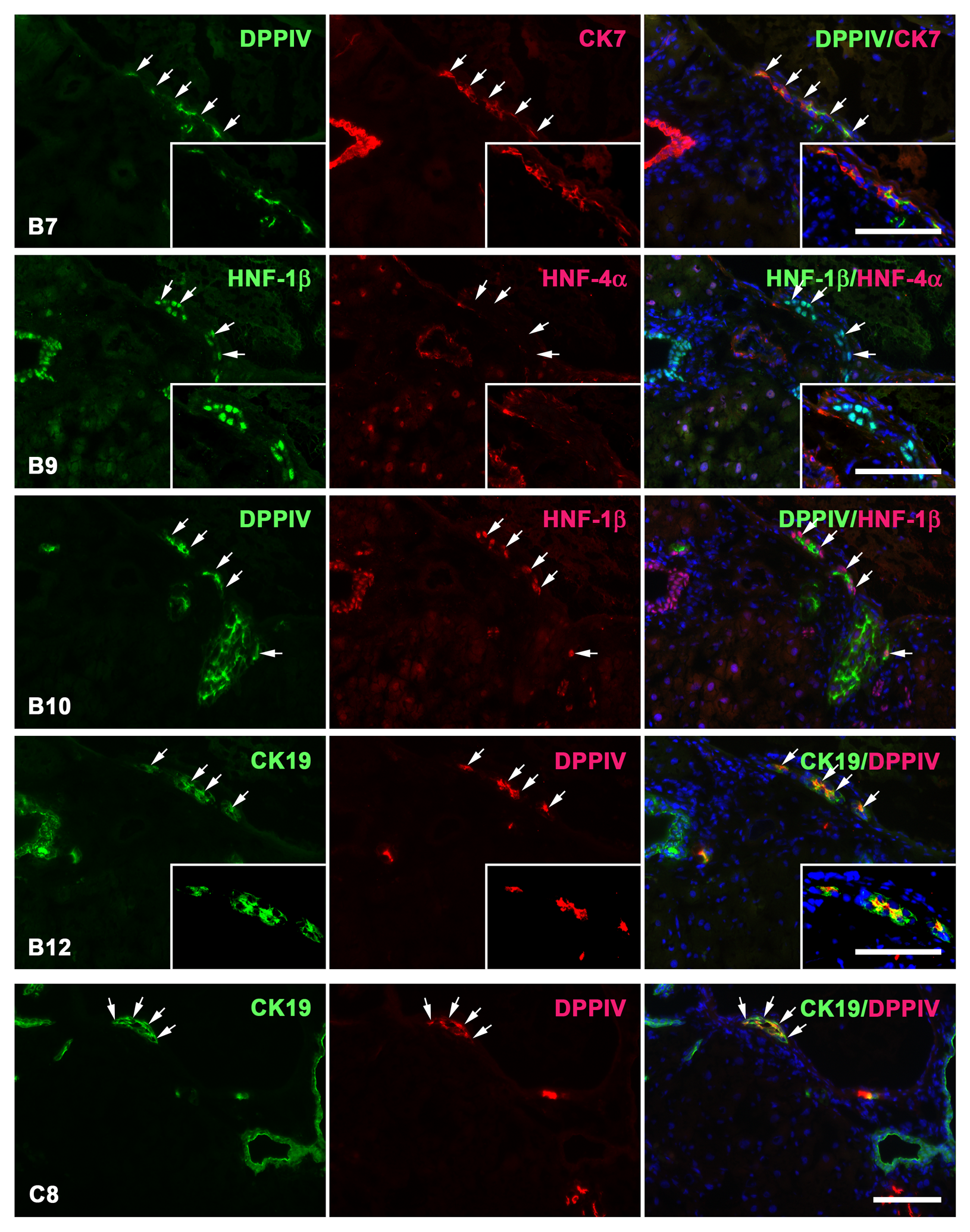

Supplement: S3 Fig — Shown are original single color and merged images of Fig 2B7 DPPIV(green)/CK-7(red), 2B9 HNF-1β(green)/HNF-4α(red), 2B10 DPPIV(green)/HNF-1β(red), 2B12 CK-19(green)/DPPIV(red), and 2C8 CK-19(green)/DPPIV(red). (Original magnification: 200x; Scale bars: 100 μm.). (TIF) [file pone.0134327.s003.tif]

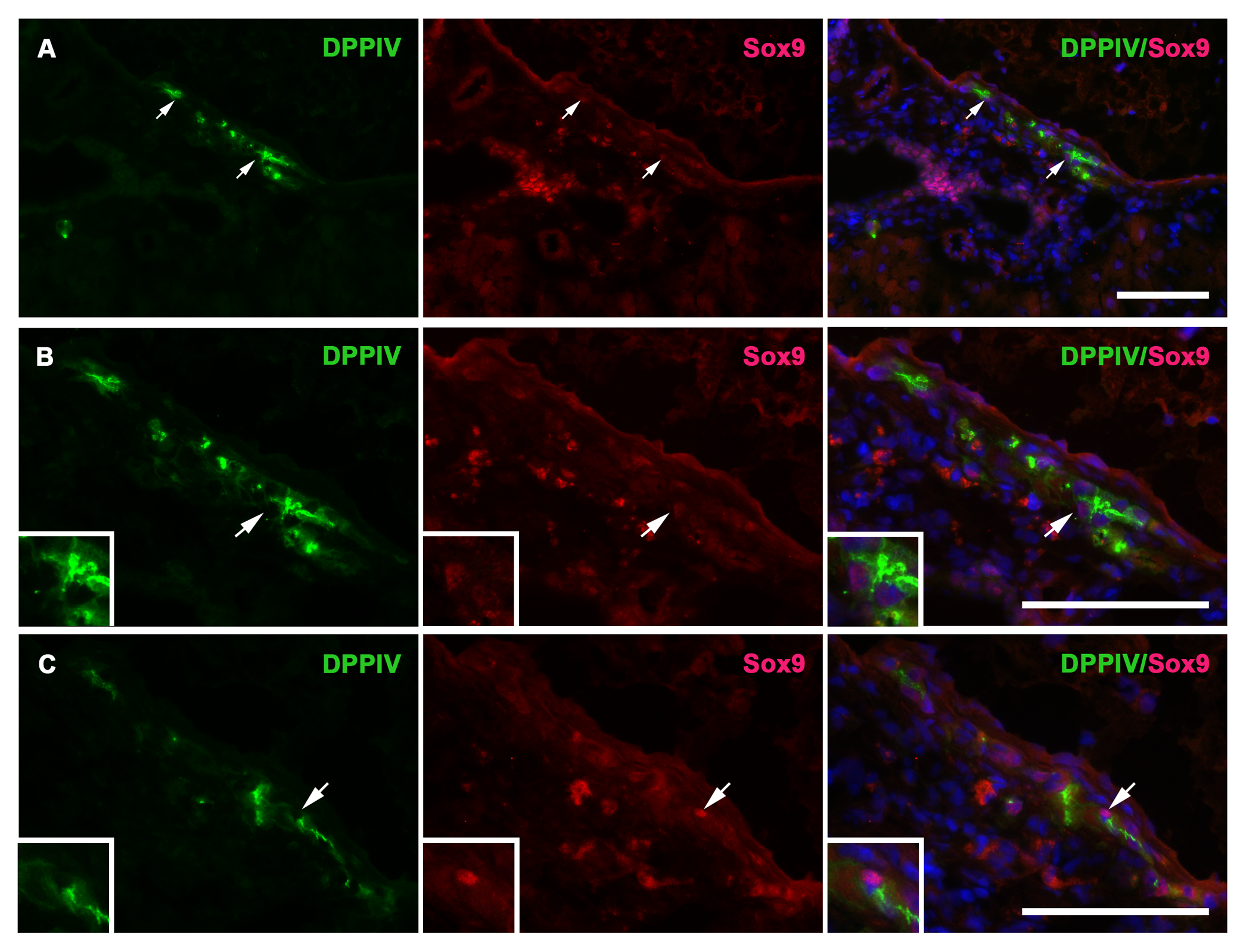

Supplement: S4 Fig — Shown are representative figures of double immunofluorescence staining for DPPIV (green)/Sox9 (red) in serial sections in R+DAPM+D-gal treated liver at 2 weeks after hepatocyte transplantation. Transplanted DPPIV-positive cells express Sox9 (arrow) at 2 weeks after hepatocyte transplantation in R+DAPM+D-gal treated liver. (Original magnification: A, 200x; B, C, 400x; Scale bars: 100 μm.). (TIF) [file pone.0134327.s004.tif]

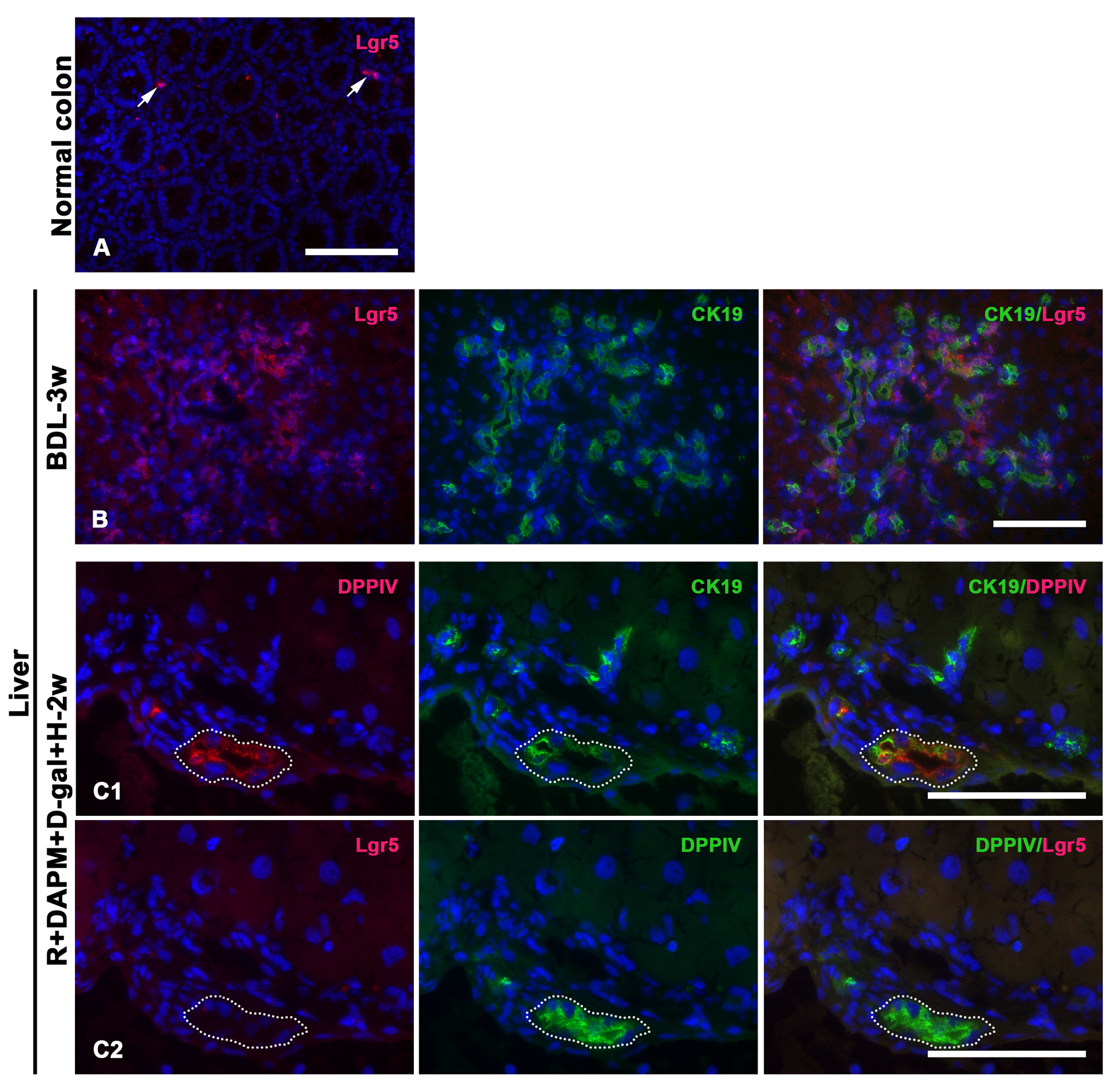

Supplement: S5 Fig — Shown are representative figures of (A) Lgr5(+) (arrow) staining in normal colon (positive control), (B) dual immunofluorescence staining for CK-19(+)/Lgr5(+) in bile duct ligation liver (BDL) at 3 weeks (positive control), and (C) dual immunofluorescence stainings for CK-19(+)/DPPIV(+) and DPPIV(+)/Lgr5(-) in R+DAPM+D-gal treated liver sections at 2 weeks after hepatocyte transplantation. Transplanted DPPIV-positive cells did not express Lgr5 during their conversion into BECs in R+DAPM+D-gal-treated livers. (Original magnification: A, B, 200x; C, 400x; Scale bars: 100 μm.). (TIF) [file pone.0134327.s005.tif]

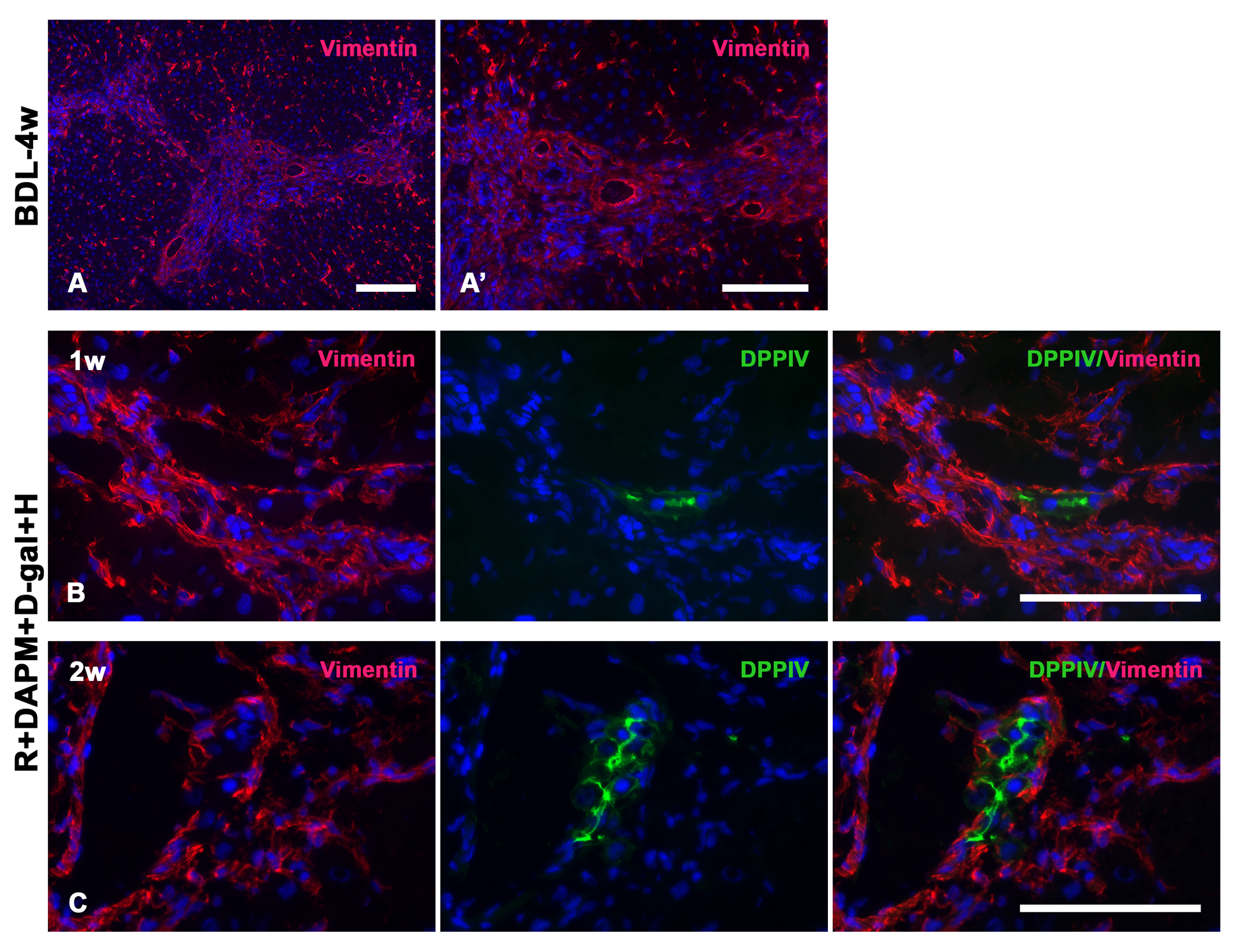

Supplement: S6 Fig — Shown are representative figures of vimentin staining in bile duct ligation liver (BDL) at 4 weeks (positive control) and of dual immunofluorescence stainings for DPPIV (green)/vimentin (red) in R+DAPM+D-gal treated liver sections at 1 and 2 weeks after hepatocyte transplantation. (Original magnification: A,100x; A’, 200x; B, C, 400x; Scale bars: 100 μm.). (TIF) [file pone.0134327.s006.tif]
